# How Informal Carers Support Video Consulting in Physiotherapy, Heart Failure, and Cancer: Qualitative Study Using Linguistic Ethnography

**DOI:** 10.2196/51695

**Published:** 2024-05-31

**Authors:** Lucas Martinus Seuren, Sara Shaw

**Affiliations:** 1 Nuffield Department of Primary Care Health Sciences University of Oxford Oxford United Kingdom; 2 Institute for Better Health Trillium Health Partners Mississauga, ON Canada

**Keywords:** delivery of health care, remote consultation, carer, telemedicine, videoconferencing, language, linguistics, gestures, physiotherapy, heart failure, care, patient care, feasibility, safety, communication, mobile phone

## Abstract

**Background:**

Informal carers play an important role in the everyday care of patients and the delivery of health care services. They aid patients in transportation to and from appointments, and they provide assistance during the appointments (eg, answering questions on the patient’s behalf). Video consultations are often seen as a way of providing patients with easier access to care. However, few studies have considered how this affects the role of informal carers and how they are needed to make video consultations safe and feasible.

**Objective:**

This study aims to identify how informal carers, usually friends or family who provide unpaid assistance, support patients and clinicians during video consultations.

**Methods:**

We conducted an in-depth analysis of the communication in a sample of video consultations drawn from 7 clinical settings across 4 National Health Service Trusts in the United Kingdom. The data set consisted of 52 video consultation recordings (of patients with diabetes, gestational diabetes, cancer, heart failure, orthopedic problems, long-term pain, and neuromuscular rehabilitation) and interviews with all participants involved in these consultations. Using Linguistic Ethnography, which embeds detailed analysis of verbal and nonverbal communication in the context of the interaction, we examined the interactional, technological, and clinical work carers did to facilitate video consultations and help patients and clinicians overcome challenges of the remote and video-mediated context.

**Results:**

Most patients (40/52, 77%) participated in the video consultation without support from an informal carer. Only 23% (12/52) of the consultations involved an informal carer. In addition to facilitating the clinical interaction (eg, answering questions on behalf of the patient), we identified 3 types of *work* that informal carers did: facilitating the use of technology; addressing problems when the patient could not hear or understand the clinician; and assisting with physical examinations, acting as the eyes, ears, and hands of the clinician. Carers often stayed in the background, monitoring the consultation to identify situations where they might be needed. In doing so, copresent carers reassured patients and helped them conduct the activities that make up a consultation. However, carers did not necessarily help patients solve all the challenges of a video consultation (eg, aiming the camera while laying hands on the patient during an examination). We compared cases where an informal carer was copresent with cases where the patient was alone, which showed that carers provided an important safety net, particularly for patients who were frail and experienced mobility difficulties.

**Conclusions:**

Informal carers play a critical role in making video consultations safe and feasible, particularly for patients with limited technological experience or complex needs. Guidance and research on video consulting need to consider the availability and work done by informal carers and how they can be supported in providing patients access to digital health care services.

## Introduction

### Background

Video consulting has become an established health care service model since the outbreak of the COVID-19 pandemic [1]. Video consultations have been shown to be safe and effective in a range of clinical settings [2-6]. Patients and clinicians have largely reported positive experiences, particularly in secondary and tertiary care [7-10], with some patients even preferring video consultations over face-to-face consultations, especially for follow-up appointments and where a trusted relationship with the provider is already in place [11]. Given the policy push for remote health care services to continue beyond the COVID-19 pandemic [12-14], it is clear that video consulting is here to stay. However, significant concerns remain around when video consulting is feasible and appropriate (eg, for which patients and in which clinical settings) [15-17]. Some patients still do not have access to the necessary technology (ie, smartphone, tablet, or computer and high-quality internet) [18,19] and they may also lack the experience, confidence, or ability to use it for a medical consultation [20-22]. In these situations, carers, either professional or informal (eg, family and friends), can provide assistance [23,24].

There is extensive literature on video consulting, documenting the benefits and challenges for patients and clinicians [5]. However, very few studies address how informal carers participate in video consultations [25,26]. This is surprising, given that informal carers play an important role in patient care. Informal carers, usually family or friends, “[provide] unpaid care and support to a family member, friend or neighbour who is disabled, has an illness or long-term condition, or who needs extra help as they grow older” [27]. In the United Kingdom, approximately 6 million people provide unpaid care, many of whom play a vital role in coordinating and supporting care received by the person they care for [28,29]. Therefore, it is important to understand the role they play in supporting and delivering video-consulting services.

Contemporary health care systems and policy makers have been pushing a transition to patient-centered or person-centered care, that is, care that is “respectful and responsive to individual preferences, needs and values” [30]. However, person-centered care has often been taken to only mean patient-centered care. Guidelines do not always address family or carers, and where they do, they merely highlight that practitioners must involve carers in patient care, for example, by asking them to clarify the patient’s wishes [31]. In other words, the focus in person-centered care is still on the patient. Nevertheless, informal carers play a central role in the delivery of care, supporting patients (to varying degrees and in varying situations) with their needs and care. Carers may deliver up to 90% of the care and support for patients in the community [32]. Therefore, it is potentially important for guidance on video consultations to take carers, and the support of carers, into account. Given that the work done by carers can cause a significant mental and physical strain [33], practitioners and policy makers need to consider the preferences, needs, and values of patients and carers.

Health communication research has shown that carers sometimes play an active role in making in-person consultations work: carers may speak on behalf of the patient (eg, to provide additional medical or other information for children or patients who lack capacity), alongside the patient (eg, when planning a next appointment), or with the patient (eg, to help them answer questions about their medication) [34-36]. However, having a carer copresent, that is, physically with the patient in the consultation, can be challenging as patients, clinicians, and carers report that they have trouble managing turn-taking [37]. This raises questions regarding when the carer is able to talk, what they can talk about, and how they can determine this.

Participation problems may be more pronounced in video consultations. From research outside the health care setting, we know that it can be difficult for carers to facilitate a conversation over video [38]. The camera restricts the field of view, and generally, only 1 person is visible at a time on each end [39]. The clinical context adds additional challenges, with participants having to manage new interactional skills (eg, how to begin a video consultation) and accomplish activities that are constrained by the lack of physical copresence (eg, conducting a physical examination) [5,40,41].

To date, only 1 study has investigated how the constraints of technology affect communication in health care where informal carers are copresent, focusing on postoperative cancer consultations in the Netherlands and showing that carers often remain offscreen and do not actively participate, and when they do, they mostly talk to the patient [42]. Several other studies have investigated how professional carers (eg, copresent nurses or primary care physicians) participate in video consultations, with a focus on how these professional Despite their crucial role in health care delivery, informal carers have not yet benefited from the advancements made in this field [29].

### Objectives

Overall, there is a need to understand how informal carers support video consultations when they are copresent with the patient. This study focuses on how informal carers support patients and clinicians during video consultations. Our focus is on the *work* (either interactional, clinical, or technological) that informal carers do to make video consultations work to provide key insights into how they affect the feasibility of video consulting. To support our analysis, we compared the consultations where informal carers provided support and the reflections of participants in subsequent interviews with consultations where patients were alone and the reflections of those participants.

## Methods

### Study Design

We conducted a qualitative, multimethods study using Linguistic Ethnography, which combines ethnographic approaches (ie, observation and interviews) with the close inspection of interactional data [43]. We used *ethnography of communication* [44] to guide our understanding of how the context of video consultations (eg, restricted visual field) may shape the ways in which patients, carers, and clinicians communicate over video. We combined this with conversation analysis, an inductive method that investigates the turn-by-turn construction of a conversation, to understand the communication practices (verbal and nonverbal) that make up a video consultation [45]. Combining these methods enabled us to show how the interactions in video consultations shape, and are shaped by, the wider sociocultural and clinical contexts (eg, established clinical routines, policy, and technology in use) [46].

For this study, we conducted secondary analysis of qualitative data that were previously collected for 3 separate studies on video consultations in different clinical settings across 4 National Health Service clinics in the United Kingdom (1 in Oxford and 3 in London):

- Supporting Consultations in Remote Physiotherapy (SCiP; 2021-2022) was funded by the National Institute for Health Research to investigate the feasibility and practicalities of video consultations for physiotherapy.
- Virtual Online Consultations: Advantages and Limitations (VOCAL; 2015-2017) was funded by the National Institute for Health Research and investigated (gestational) diabetes and cancer.
- Oxford Telehealth Qualitative Study (OTQS; 2015-2017) was funded by the Wellcome Trust to investigate the feasibility of video consulting in a specialist nurse service for patients with heart failure.

Data were chosen for convenience, having been collected as part of research studies that had already been conducted by members of the larger research team and available for secondary analysis [47,48].

### Data Collection

We analyzed all 52 video recordings of video consultations that were recorded across the 3 studies. Data for VOCAL and OTQS were collected from 2015 to 2017 (refer to the study by Shaw et al [5] for an overview), and data for SCiP were collected from 2021 to 2022 (refer to the study by Seuren et al [47] for an overview).

In all 3 studies, recruitment was done based on convenience. For VOCAL and OTQS, which took place before the COVID-19 pandemic when video consulting was still relatively unfamiliar, patient participants were recruited in collaboration with clinical staff to ensure that patients were suitable to have a video consultation. The aim was to create a sample with a range of experiences with video consultations, “seeking maximum variety in clinical, ethnic and personal circumstances.” Patients were initially contacted by their clinician, after which the research team sent out an invitation letter [5]. For SCiP, data collection took place between August 2021 and March 2022, during the COVID-19 pandemic. Initially, clinicians reached out to any patient who had an upcoming appointment by video. Those who showed an interest in the study were subsequently contacted by a member of the research team to explain the details of the study [47]. For all studies, exclusion criteria were the inability to give informed consent and comorbidity preventing participation. For VOCAL and OTQS, additional exclusion criteria were no 3G internet access at home and lack of familiarity with technology [5].

Video consultations for VOCAL and OTQS were recorded using small digital camcorders (Sony Handycam DCR-SR72; Sony Corporation) and a handheld iPad (Apple Inc), combined with a commercially available screen-capture software tool (ACA Systems), which was run directly from an encrypted USB memory stick. Whenever feasible, both the patient’s and the clinician’s end of the consultation had been recorded, capturing the consultations and the context in which they took place. The total data set from VOCAL and OTQS consisted of 37 video recordings and transcripts of the video consultations (cancer: 12/37, 32%; diabetes: 12/37, 32%; heart failure: 7/37, 19%; and gestational diabetes: 6/37, 16%), 35 transcripts of semistructured interviews conducted with staff and 26 transcripts of semistructured interviews conducted with patients involved in these consultations (Table 1) [5].

Video consultations for SCiP were recorded by the clinical team in the 2 National Health Service Trusts using the built-in recording option in Microsoft Teams (Microsoft Corp). The total data set consisted of 15 video recordings and transcripts of video consultations (neuromuscular rehabilitation: 5/15, 33%; long-term pain: 1/15, 7%; and orthopedics: 9/15, 60%), 15 transcripts of semistructured interviews with patients and 7 transcripts of semistructured interviews with clinicians involved in these consultations (Table 2) [47].

**Table 1:** Participant data from the Virtual Online Consultations: Advantages and Limitations study and Oxford Telehealth Qualitative Study (n=37).

Clinic	Total recordings, n (%)	Gender, n (%)	Age (years), median (IQR)	Ethnicity, n (%)
Diabetes	12 (32)	Male: 5 (42) and female: 7 (58)	23 (22-35)	Asian Bangladeshi: 1 (8), Asian Indian: 3 (25), Black Caribbean: 1 (8), White British: 5 (42), and White Other: 2 (17)
Gestational diabetes	6 (16)	Female: 6 (100)	34 (30-35)	Asian Bangladeshi: 1 (17), Asian Other: 3 (50), Black Caribbean: 1 (17), and White British: 1 (17)
Cancer	12 (32)	Male: 4 (33) and female: 8 (67)	74 (60-77)	Asian Indian: 1 (8), Black Caribbean: 1 (8), White British: 9 (75), and White Other: 1 (8)
Heart failure	7 (19)	Male: 3 (43) and female: 4 (57)	67 (56-76)	White British: 7 (100)

**Table 2:** Participant data from the Supporting Consultations in Remote Physiotherapy study (n=15).

Clinic	Total recorded, n (%)	Gender, n (%)	Age (years), median (IQR)	Ethnicity, n (%)
Orthopedics	9 (60)	Female: 8 (89) and male: 1 (11)	35 (29-57)	White British: 7 (78), White Irish: 1 (11), and White Mauritian: 1 (22)
Neurology (neuromuscular)	5 (33)	Female: 1 (20) and male: 4 (80)	41 (40-47)	White British: 5 (100)
Long-term pain	1 (7)	Female: 1 (100)	20^a^	White Scottish 1 (100)

^a^There was only one participant; hence, there is no IQR.

### Analysis

An initial exploration of the 52 recorded video consultations across all 3 studies showed that informal carers performed a range of seemingly vital tasks in some (but not all) video consultations (12/52, 23%; eg, holding the tablet and laying hands on the patient). This raised questions about the role of carers in video consultations. We collected all instances in our video data where carers were involved at any point during a video consultation and corresponding interview data in which participants in these video consultations reflected on the work carers do. As a routine practice in conversation analysis [49,50], we then built “collections” of similar cases [51], organizing the data based on the type of work done by carers. To further refine our analysis, we compared our findings with consultations where no carer was present (40/52, 77%), combining researcher observations of potentially risky situations (eg, an older patient nearly fell) with clinician reflections on these consultations to identify cases where the lack of a copresent carer might have negatively affected the quality of care. On the basis of these collections, we then analyzed the qualitative interviews deductively using thematic analysis [52]. Themes were identified based on our analysis of the consultations and used deductively to analyze the interviews. We examined how participants talked about the 3 key themes, aiming to discern whether participants’ reflections were in line with our findings of the consultations (eg, when and why do patients require assistance with technology) or whether they offered complementary (eg, additional work done by informal carers outside of the consultation) or even contradicting viewpoints (eg, informal carers not being able to offer support). Our analysis focused on the conversation analysis of the consultations, with supporting reflections from the participants.

As all data were selected for convenience, the consultations that involved a carer and those that did not involve a carer were not matched regarding, for example, clinical context, patient demographics, or type of technology used.

We transcribed all video consultations orthographically and subsequently used established conventions for verbal and nonverbal communication [53,54] for the data in our collections. This is a routine practice in conversation analysis and, for this paper, enabled us to track how and why carers assist in video consultations. In the Results section, we present simplified extracts from transcripts, providing orthographic transcripts complemented with notations for silence and overlapping talk to maintain legibility. We added screengrabs to allow readers to appreciate the context of consultations and how participants use their bodies and material objects (eg, how they move and hold a tablet). All interviews were transcribed orthographically. We extracted screengrabs using Adobe Premiere Pro 2023 (Adobe Inc), adding a video filter and facial blur to deidentify participants. Subsequently, we combined these screengrabs with the transcript in Adobe InDesign 2023 (Adobe Inc) and exported these at 600 dots per inch to generate the figures.

### Ethical Considerations

All studies received ethics approval for detailed analysis of video recordings of video consultations and audio recordings of interviews. VOCAL was approved by the National Research Ethics Committee London-City Road and Hampstead in December 2014 (14/LO/1883), OTQS by the South Central-Berkshire Research Ethics Committee in September 2015 (15/SC/0553), and SCiP by the East Midland-Nottingham 1 Research Ethics Committee in April 2021 (21/EM/0082). All participating staff and patients provided their informed consent to be audio and video recorded during consultations and interviews and for the data to be used for research purposes, including secondary analysis.

Patients were initially approached by a member of their clinical care team. After signaling an interest in the study, the patient’s contact information was forwarded to a member of the research team. The author provided the patient with an information sheet to review. After providing an opportunity to ask questions, patients were asked if they wanted to participate, and if they agreed, they were asked to sign the consent form. For VOCAL and OTQS, patients provided consent during an in-person conversation with a member of the research team. For SCiP, to comply with infection control procedures during the COVID-19 pandemic, patients provided verbal consent during a video call. Participants did not receive compensation for participation in any of the 3 studies.

All transcriptions were anonymized by removing identifying data and replacing names with descriptions (eg, NAME, where someone’s name is used). Participants consented to the analysis of the raw (ie, recognizable) video data. For publication, video data were anonymized using a visual filter and blur effect in Adobe Premiere Pro 2023.

## Results

### Main Findings

Of the 52 video consultations in our data, 12 (23%) involved a copresent carer: 8 (67%) with patients with cancer, 3 (25%) with patients with heart failure, and 1 (8%) with a patient consulting for physiotherapy. None of the patients with gestational diabetes had a copresent carer. In these 12 consultations, we identified three main types of work that carers performed: (1) facilitating the use of the technology, (2) helping the patient hear or understand what the clinician said, and (3) assisting the patient with and performing parts of the physical examination. Carers performed these tasks through the use of verbal and nonverbal communication strategies, as seen in the data extracts, screengrabs, and participants’ reported experiences in the following sections. Furthermore, we found that in 10% (5/52) of the consultations the patient did not have a carer copresent, but either the patient or clinician expressed concerns regarding safety during the consultation (eg, a patient saying, “I’m not sure if I’ll be able to get back up again”) or the clinician, during the interview afterward, commented that they felt they might have put the patient in an unsafe situation.

### Facilitating the Use of Technology in Video Consultations

Informal carers facilitated the use of technology for video consultations in 2 ways: they provided patients access to the technology, and their presence and perceived expertise provided patients with confidence and reassurance for using the technology.

In our data, some patients (5/52, 10%) either did not have the technology or had never used it for video chat. Therefore, they relied on carers to set up, and sometimes provide, the technology. This facilitation involved activities such as the carer bringing a tablet for the patient to use, registering a Skype (Skype Technologies) account, adding the clinician as a contact on Skype or FaceTime (Apple Inc), talking to the clinician beforehand regarding any practicalities, and explaining to the patient what to expect from the video consultation. For the patients who lacked experience with video-mediated communication, carers provided a sense of reassurance if something went wrong or if there were difficulties. This was evident both in how the informal carers acted in the consultations and how they discussed their experiences during the interviews. An older patient explained before her oncology consultation that she only agreed to a video consultation if her husband would be there:

First uh, I was a bit uh, I said uh, if he’s here it’s fine. I haven’t got any problem.

Another older patient stated after her heart failure consultation that, while she could learn to use the technology, she relied on her daughter being there and would not have been able to do it on her own:

Patient: that’s what I really think, that for me,... it’s easy. Because I don’t have to sit here and think, what if I do something wrong?

Carer: no

Patient: for people, old people on their own, entirely different.

Carer: yeah. it is entirely different.

Patient: And I would not be able to do it on my own. ... I wouldn't have the confidence.

During consultations, we found that carers often facilitated the use of technology while being silent (ie, nonspeaking) and offscreen. This involved carers performing 3 types of background activities that allowed the patient to consult with the clinician via video: they handled the “preopening,” the work people do before they start a video consultation [55]; they handled the camera allowing the patient and clinician to adequately see each other; and they made sure that the patient and clinician could hear each other.

In 8 (67%) of the 12 consultations, carers took care of the “preopening” [55]: they set up the technology, logged in, and answered the call from the clinician when using a program such as Skype or FaceTime. Then, the patient took over when the consultation started.

**Figure 1:**
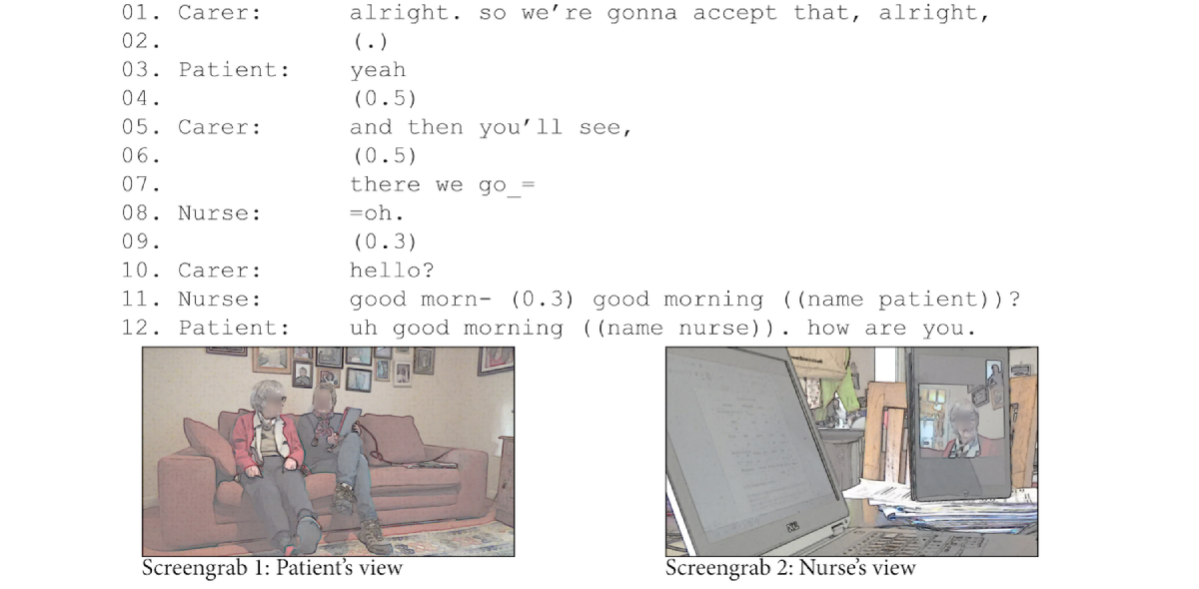
Carer helping the patient at the start of a video consultation.

In the example in Figure 1, the patient had never used FaceTime before and did not own a video communication technology (eg, a smartphone or tablet). The carer brought a tablet with her, signed into FaceTime, and held it ready for use. When the clinician called via video, the carer explained to the patient that they would accept the call (line 1). Then, she swiped to answer, pointed out to the patient when the connection was established (line 7) and answered the video call with a "hello, conveying to the clinician that the connection had been established and they were ready [56]. The carer stayed out of the frame (refer to screengrab 2 in Figure 1) and hence out of the interaction [42,57], allowing the patient to conduct her consultation while still remaining available in the background.

In 7 (58%) of the 12 consultations, carers did additional background work that enabled patients to talk to their clinician. This included making sure that the patient and clinician could adequately see and hear each other, for example, by acting as a cameraperson: positioning the technology and framing the patient throughout the consultation [38] to maintain a “talking heads” configuration for the patient and clinician [39], a setup in which both participants are visible from the shoulders up. In 71% (5/7) of these consultations, the carer held a tablet or smartphone, moving this to frame the patient while remaining outside the frame themselves. In 29% (2/7) of these consultations, the patient used a desktop PC, so the carer moved the patient instead of the technology. 

**Figure 2:**
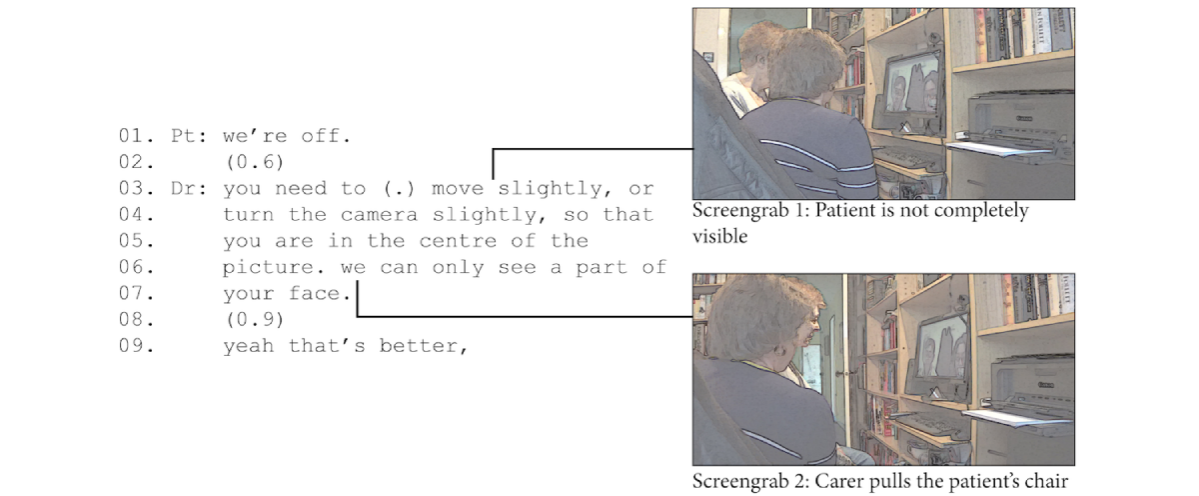
Carer helping to make the patient visible.

In the example in Figure 2, from the start of an oncology consultation, the patient was at the left edge of the field of view of the camera and only half of her face was visible to the clinician. As soon as the physician told the patient to “move slightly” (line 3), the carer turned toward the patient and began to pull their chair. At the point where the physician completed his request (line 8), the patient was visible in the center of the screen. Our recording of the clinician’s end does not capture the screen. However, on the screen on the patient’s side, we can see that initially only the right half of her face is visible, and the carer then adjusts the chair so that the patient is centered and fully visible.

**Figure 3:**
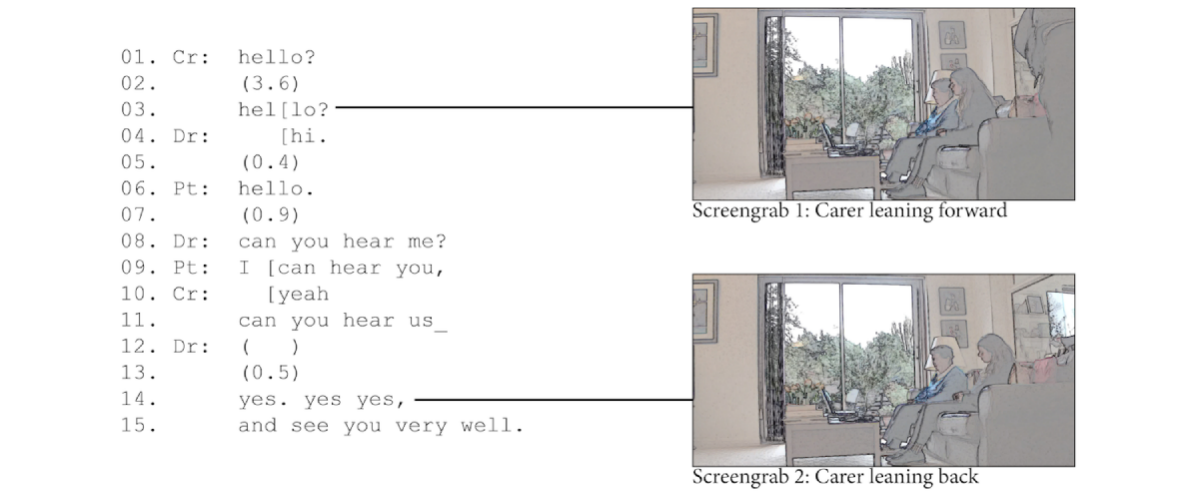
Carer checking the connection at the opening of a video consultation.

In 4 (33%) of the 12 consultations, the carers acted as a technological facilitator to ensure the audio and video were working. Carers did most of this work at the start of the consultation. This was the first point where participants could determine whether the sound and video were working. In the example in Figure 3, the carer answered the clinician’s call when he appeared on screen by saying “hello” (line 1), but the clinician did not respond. The carer treated this silence as indicating a problem: she said “hello” again but this time with a more *questioning* intonation (a strong rising pitch on the “o”), a typical communication strategy for testing if someone can still hear [58].

After the clinician had said “hi” (line 4), he asked whether the patient (and carer) could hear him (line 8). The patient and carer confirmed (lines 9-10), and the carer checked whether the clinician could hear them. In other words, before the consultation began, the carer and clinician ensured that the technology was working and that the patient and clinician could see and hear each other. It was only when the physician had confirmed (line 14), that the consultation proceeded in a usual manner. At this point, the carer faded into the background.

**Figure 4:**
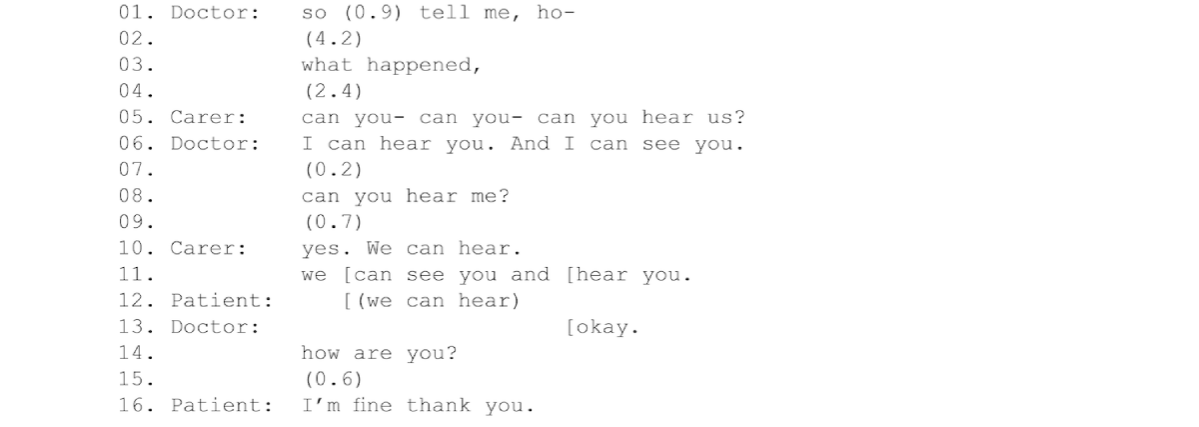
Carer troubleshooting the connection problem.

Staying largely in the background (and so invisible to the clinician), carers typically maintained an active role, helping to address any problems (eg, lost connection or microphone on mute) that arose during the consultation. In these instances, carers temporarily became active participants while fixing the problem. In the example in Figure 4, the physician asked the patient “how are you.” However, a technical disruption occurred and his turn was cut off after “ho.” After a few seconds of silence, the physician said, “what happened” (line 3), taking the lack of response by the patient as indicative of a problem. It was the carer who then switched to become an active coparticipant, asking if the physician could hear them (line 5). Once all parties had established that they could see and hear each other, the physician acknowledged (line 13) [59] and repeated the question.

Overall, carers in our data made video consultations feasible by facilitating the use of technology. Much of this work involved carers moving from being coparticipants to listening in the background, often unobservable to the clinician. They did this either by self-selecting to respond to a clinician’s question (Figures 3 and 4) or by being selected by the clinician to answer a question. After responding, they would visually move out of the screen, or at least, no longer respond or take turns.

### Making the Interaction Work in Video Consultations

Patients in our study occasionally had problems with hearing or understanding the clinician (eg, due to soft or distorted sound). Such problems happen routinely in any form of conversation [60], and people have a large array of *repair* strategies to fix them [61,62]. Normally, when trouble arises, recipients ask the speaker to repeat or clarify (part of) their turn (eg, by repeating the part of the turn they did hear or using exclamations such as “sorry,” “what,” or “huh” [63-65]). During in-person consultations, if patients have problems, they can ask the clinician to clarify [66].

**Figure 5:**
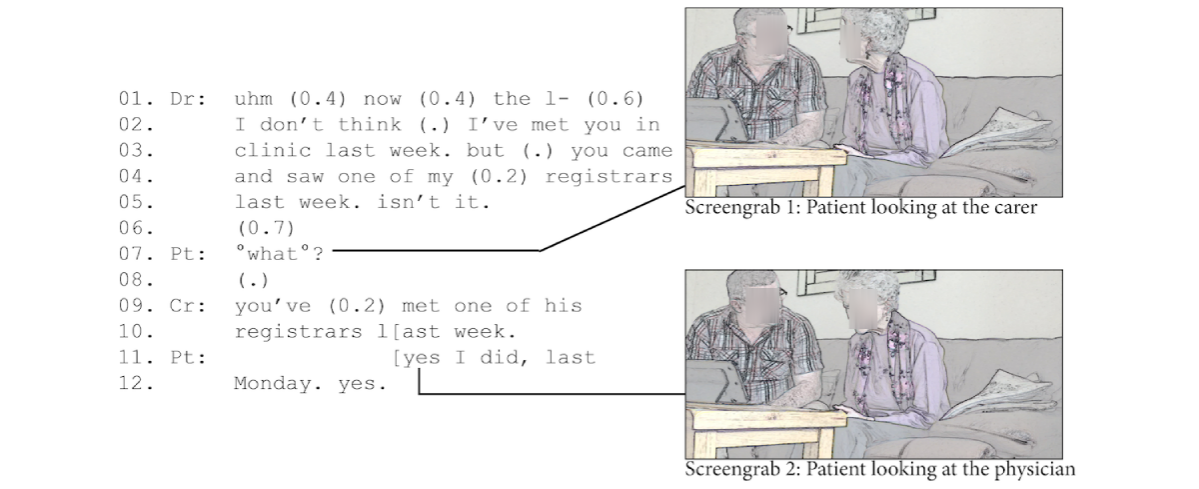
Carer repeating the clinician’s question.

In our video consultation data, we found that 25% (3/12) of the patients relied on their carer to help them hear or understand the clinician’s talk (in all 3 consultations, the quality of the call was problematic, eg, low volume and distortions). The example in Figure 5 illustrates how carers perform this type of interactional repair. In lines 1 to 4, the physician checked that the patient had seen one of his registrars the week before at an in-person consultation. At this point, the volume was low, making it hard to hear. Moving into the physician’s turn, the patient started squinting (refer to screengrab 1 in Figure 5), indicating she had a problem. When the physician finished his question, the patient remained silent for 700 milliseconds (a substantial amount of time, given the usual response time for face-to-face interaction being 0-200 milliseconds [67]), indicating difficulty [68]. Instead of answering, the patient turned to the carer (refer to screengrab 2 in Figure 5), softly asking “what?” (indicated with the degree symbols) and expecting the carer to perform an interactional repair on the physician’s question. The carer (offscreen) repeated the physician’s verification question in line 8. Once the patient could answer, she started to nod, turned her gaze toward the physician (line 9), and answered (line 11) loudly, thereby making clear her response was now directed to the physician.

While this was a brief interaction, the carer in this example played a crucial role in the successful communication between the physician and the patient. The patient mobilized the carer to help her hear what the physician said. Akin to an interpreter, the carer “animated” the physician’s talk [57]. Similar examples using indirect communication (eg, physically turning to the carer when something was unclear) were evident across our data set, where patients sought help from carers to enable repair and continuation of the interaction.

### Making Physical Examination Possible in Video Consultations

The final area where carers made a vital contribution to video consultations was during physical examinations. The inability of clinicians to lay hands on the patient is one of the main concerns among clinicians and patients about video consultation [69-71]. Instead, patients have to describe and show their body and, where available, use their own devices such as oximeters (a device that people clip onto their finger to measure their blood oxygen saturation and heart rate) [40,72].

Carers supported remote physical examinations in 8 (67%) of the 12 video consultations in our data. This included helping to make the relevant parts of a patient’s body visible, acting as the clinician’s hands to perform tactile examinations and providing visual assessments, and assisting the patient with operating equipment such as blood pressure meters. Support was typically for patients who were frail, in cases where they were either unable to bend over (eg, due to the nature of their condition) or unable to move their tablet or laptop at the same time as moving their body (a complex sociotechnical task that was particularly challenging for those experiencing chronic illness) [72].

**Figure 6:**
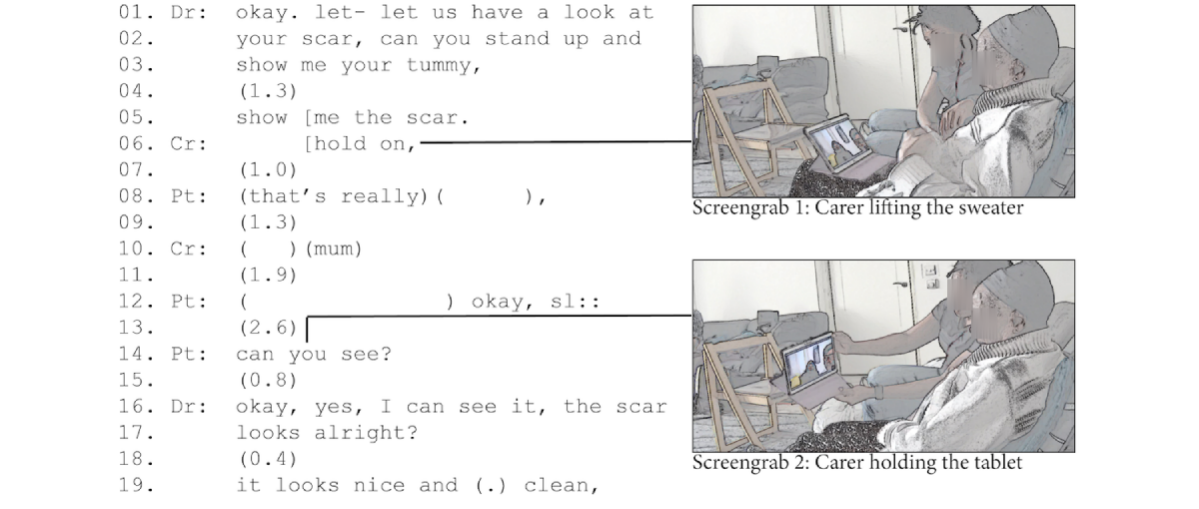
Carer assisting in making the patient’s scar visible.

Figure 6 illustrates how carers can play a vital role in the feasibility of a physical examination. The patient had recently undergone surgery to remove a tumor and had complained to the physician about pain in her abdomen around the scar. The physician asked to examine the scar, requesting her to stand up (lines 1-3). The patient did not respond to this request. Instead, she waited for the carer to help out. After 1.3 seconds of silence, the physician made his request again, but at the same time, the carer said “hold on.” Then, the carer helped the patient lift her sweater and aimed the camera toward the scar, allowing the physician to perform a visual assessment (lines 16-19).

In the example in Figure 6, the role of the carer was crucial for making the physical examination work. With limited physical capacity (and technological literacy), the patient was unable to hold the tablet and show the clinician her abdomen. It was only with the help of her carer that she could provide a sufficiently clear view for the physician to perform a visual assessment remotely.

**Figure 7:**
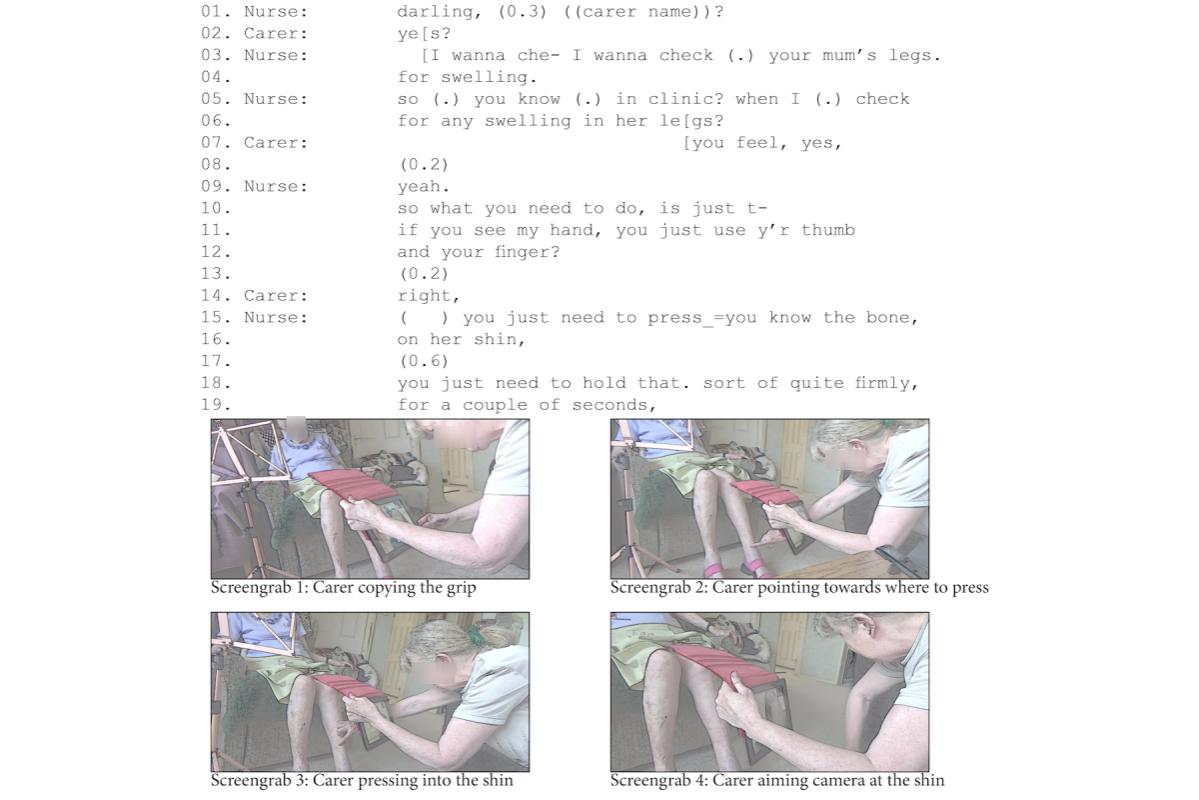
Carer assessing for edema.

At times, clinicians relied on a carer to lay hands on the patient on their behalf during video consultations. In the heart failure consultations—both routine follow-up consultations—the specialist nurse wanted to check whether the patients had fluid build-up (edema) in their legs and ankles by pressing their thumb on the patient’s leg and check whether this leaves an indentation. Carers played a vital role in performing these remote assessments, which involved patients who were frail, with restricted mobility and breathlessness, and for whom moving could cause severe discomfort [72]. Figure 7 illustrates an example in which the patient had just measured her blood oxygen saturation with an oximeter. Then, the nurse addressed the carer directly, announcing that she wanted to check the patient’s legs (lines 1-4). Depicting how the carer should hold her hand (lines 11-16) [72,73], she explained how to press (lines 18-19). The carer followed these instructions and pressed the patient’s legs several times. Using the camera on the back of the tablet, she not only performed the examination but also did so while simultaneously monitoring what the nurse could see (refer to screengrabs 2 to 4 in Figure 7). The carer’s presence meant that the nurse was able to make a good assessment of the patient’s legs, telling the carer that “you’re doing a good job, and I can see it really clearly on the screen.”

**Figure 8:**
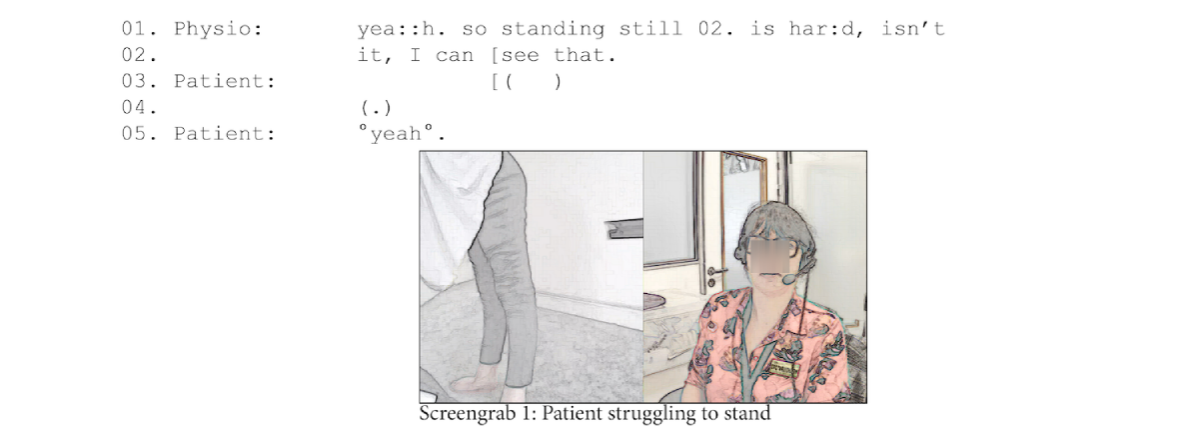
Patient struggling to stand.

In total, 2 (4%) of the 52 cases in our wider data set of video consultations flagged questions regarding the safety of physical assessment where carers were not present. Figure 8 illustrates the example of a neuromuscular physiotherapy consultation with a patient with Charcot-Marie-Tooth disease (a neurological disorder that causes damage to the peripheral nerves leading to muscle weakness and atrophy), who struggled with walking and balance. At one point, the clinician asked the patient to stand up so that she could see her walk while holding onto a wall. The patient had to push herself from the bed, had difficulty standing up without losing her balance, and had to use both hands to help herself. In hindsight, the clinician acknowledged that this may have been too difficult.

**Figure 9:**
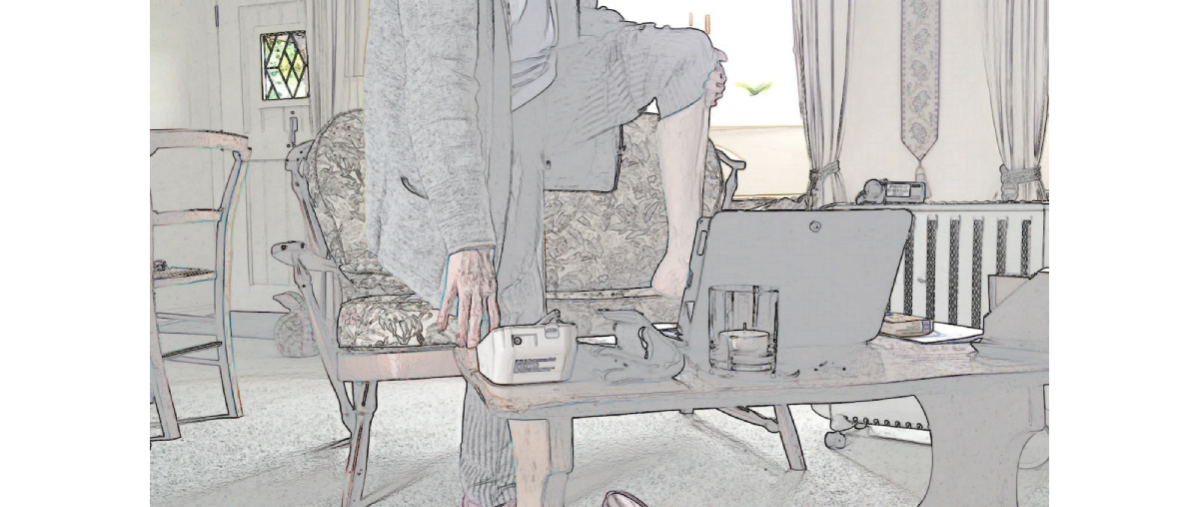
Older patient holding her leg up to be seen in the camera.

We identified a similar case in our heart failure data, where an older patient raised her leg to the camera, allowing her nurse to assess whether there was any swelling (Figure 9). The patient needed to stand and had to hold on to the chair in front of her to maintain her balance, but the uncomfortable position caused abdominal cramps and led her to drop her leg. This raised questions regarding safety while also placing limits on what was only a brief visual assessment.

**Figure 10:**
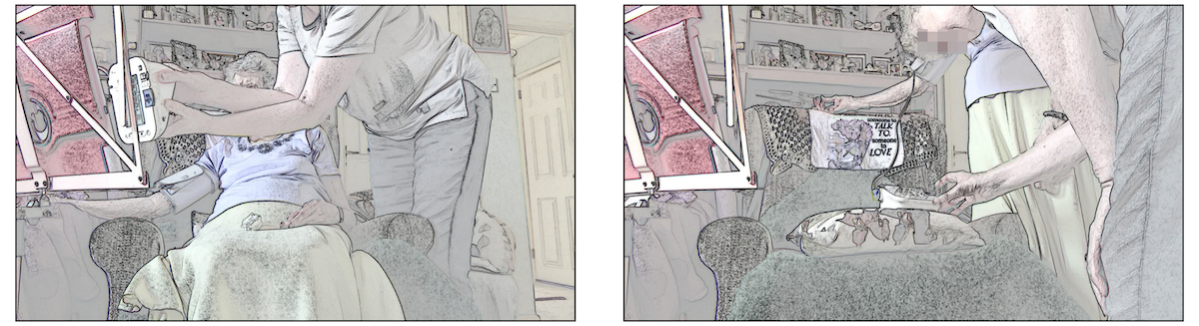
Carer operating the blood pressure monitor.

Furthermore, carers helped some patients (2/12, 10%) operate measuring devices during examination. This was particularly relevant in remote heart failure consultations, in which all 7 patients needed to measure their oxygen saturation, blood pressure, and heart rate. All 7 patients were able to use the oximeter; however, operating a blood pressure meter proved challenging for 2 patients, both experiencing frailty. In both cases, the patient’s carer placed the cuff on their arm, held the monitor up to the screen to display the results, and adjusted positions so that the patient’s blood pressure measurements could be obtained from both sitting and standing positions (Figure 10).

Remote physical examinations are complex sociotechnical tasks, involving (in our data) at least 3 people, multiple devices at both ends of the call, and a series of instructions and interactions conducted over a video consultation [72]. Hence, while carers were often needed to make physical examinations work, the assistance of a carer did not make them straightforward. In 1 consultation, video largely restricted the examination to a visual inspection. As the carer from Figure 6 reported after the consultation, with an in-person consultation, “[the patient] could probably explain more where it hurts and [the physician] could, you know, feel why it’s, you know, still tender.” Furthermore, doing the examination while making it visible to the clinician can be challenging for the carer [72]. This was summarized by the carer from Figure 1 as follows:

Well, look at me, fannying about just trying to get a picture of your leg. I mean it’s not a matchstick. I just could not picture. But it’s partly, because I’m holding it and I can’t see what I’m looking at.

## Discussion

### Principal Findings

Our findings demonstrate that, for some video consultations with some patients, informal carers play an important role in supporting setting up and running a video consultation. While most patients (40/52, 77%) in our data completed the consultation on their own, informal carers were the linchpin that made the video consultation safe and feasible, especially when the patients lacked technological literacy or experienced high frailty. We demonstrated this using recordings and observations to show 3 types of *work* that carers perform. First, they help patients use video technology by setting up everything beforehand and acting as technological support, providing patients with the confidence to even commit to using video. Second, where patients struggle to hear or understand the clinician, carers perform the interactional repair work, repeating or clarifying the clinician’s words. Third, where physical assessments are needed, carers can lay hands on the patient’s or the clinician’s behalf or assist the patient with using the technology (either video technology or examination equipment). Even where patients seemed to manage on their own, patients performed maneuvers that put them at risk of falling, and this was not always clear to the remote clinician. Copresent carers provide an important safety net, making video consultations safe and feasible.

### Comparison With Previous Research

There is an extensive body of research on the feasibility and acceptability of video consultations [6-10], which indicates that some patients may need assistance from carers [74]. However, to date, no study has investigated the work informal carers do to support video consultations. A total of 3 health communication studies have used robust methods for analyzing interaction to demonstrate how carers, whether professional or informal, can be involved in a consultation. Two (67%) of these 3 studies documented that nurses and general practitioners play an essential role in making physical examinations work when patients talk to a remote consultant [74,75]. One study on follow-up consultations after surgery showed that informal carers mostly act as bystanders: they remain invisible to the clinician and only occasionally facilitate the consultation [42].

Our study adds to this growing body of literature, demonstrating that informal carers may take a more active role than that of a bystander: in our data, they are attentive to the interaction, moving into and out of the field of view of the camera as needed; performing a range of technical, interactional, and clinical tasks; and taking a more active role depending on the needs of both the patient and clinician. Discrepancies between our findings and previous studies can be accounted for in many ways. First, as both the 3 previous studies and our study are qualitative in nature, they prioritize analytical depth, which mandates small patient samples that are not necessarily representative, and these are prone to bias in recruitment processes. Second, all studies have taken place in different geographical locations, at different points in time (eg, before or during the COVID-19 pandemic), and in different clinical settings, with patients with different sociodemographic backgrounds. While the methods may be transferable, more research is needed to appreciate to what extent the findings transfer.

The important role of carers is not limited to video consulting. For in-person consultations, research has shown that carers can be actively involved, talking about, alongside, or with the patients, to provide clinicians with relevant information [34-36]. Findings from our study extend this, demonstrating not only the other types of work that carers do to support video consultations but also how the technology shapes this work.

Videoconferencing technologies and the visual angle of webcams are designed for one-to-one conversations [39]. These aspects of technology add to the complexity of the interactional dynamics that already exist for triadic consultations (ie, involving a patient, clinician, and informal carer), where participants may struggle with turn-taking [34,35]. Because of this added complexity, video consultations have a continuously shifting *participation framework* (ie, the roles of patient, clinician, and carer as, for example, an active coparticipant of overhearer) [57], where carers move in and out of a variety of interactional and technical support roles. Depending on the situation (eg, the patient’s capacity and willingness to talk on their own behalf), carers may be expected to be more or less active coparticipants during consultations. Being offscreen makes carers less available for the clinician. They are more likely to act as overhearers [42], which can be beneficial in cases where patients wish to interact with the clinician themselves, but it may also be detrimental when patients need more continuous support. Therefore, our findings contribute to not only our appreciation of the important role of carers in the delivery of health care services but also the interactional organization of video consultations. Future research should investigate systematically how the affordances of the technology, particularly the camera’s field of view, affect the norms regarding participation, quality of care, and participant satisfaction.

### Meaning of the Study

Our findings suggest that when considering the feasibility of video consultations, some important considerations need to be taken into account. Video consulting has often only been considered a suitable service model for patients with technological competence and experience, where the goal of the consultation is expected to be relatively straightforward (eg, sharing test results and routine follow-up). However, our study shows that this unnecessarily limits to application of video for 2 reasons. First, where patients have a lack of experience with or have anxiety around technology, informal carers can help overcome technological or interactional difficulties. Furthermore, they offer reassurance, making patients comfortable with doing a video consultation. Help may not be needed, but where it would be needed, it would be available [76]. Second, where the goal of the consultation is more complex (eg, involves a physical assessment), video can still be an appropriate option if the patient has adequate support. Assessments in a video consultation often require the patient to move the camera around to frame themselves in a way that they are adequately visible to the clinician while performing movements that may be difficult for them to do safely or using devices that they are not familiar with (eg, oximeters). Copresent carers can overcome some of these challenges, for example, by taking care of the camera or laying hands on the patient, where patients are comfortable with that.

Since the outbreak of the COVID-19 pandemic, video consultations have become a more routinely used service model. While many patients and providers are moving back to in-person delivery of (health) care, hybrid service models that involve remote options, including video consultation, are likely to constitute the new normal. However, despite the routinization of video-consulting services, clinicians still have limited evidence on when they are a feasible and safe option. While the literature is growing quickly and many organizations have proposed guidelines, these often ignore the role of informal carers. Further rollout of this new service model needs to consider not only what patients themselves can do but also what informal carers can do. Given the important role that informal carers have in health care management, particularly for certain groups of patients (eg, young children, patients with high frailty, or patients who lack capacity), it is logical to assume that their role can be transferred to video-consulting models. The additional work for carers will have to be weighed against the potential benefits for each specific clinical context and each individual patient.

The importance of carers for making some video consultations work raises important questions for those providing and supporting services. Not all patients will have access to an informal carer, and those who have may not always want a carer to be present during the consultation. A systematic review found that patients are not necessarily as involved during consultations where they are accompanied by a carer, and while most patients say they appreciate having someone with them, they want to be able to decide whether a carer will be present during the consultation [77]. Patients should feel comfortable asking for their carer to leave the room at any point during a consultation. However, this might put an unnecessary burden on the patient. It may be necessary for clinicians to create opportunities to talk to the patient privately.

### Strengths and Limitations

Physiotherapy consultations in our data set were conducted during the COVID-19 pandemic, with heart failure and diabetes consultations conducted before the pandemic when video consulting was not a routine service model and few patients, carers, or clinicians had experience with it. Given the uptake and learning around video consultations during the COVID-19 pandemic, it is possible that patients involved in heart failure and cancer consultations needed more support with the technology than they would now. The prepandemic data were also likely to involve early-adopter clinicians who were supportive of video consultations as a new service model. Furthermore, participants in our data set used mainly Skype (Microsoft Corporation) and FaceTime (Apple Inc), whereas video consultations now often take place on dedicated platforms such as Teams (Microsoft Corporation), Attend Anywhere, or AccuRx. Some of these platforms affect the opening of video consultations, with patients expected to join a virtual waiting room before joining the consultation with their clinician. In addition, we focused on the positive experiences of patients and carers, without actively considering whether and when clinical staff are receptive to carer involvement. Despite these limitations, we anticipate that many of our findings are transferable to current video-consulting services. Our use of methods focused on interaction and communication has enabled us to demonstrate in detail the active role that carers played in some video consultations. While the exact role of carers may differ during and after the COVID-19 pandemic, it is highly likely that some patients (eg, older patients, those experiencing frailty, or those with multimorbidity) will continue to need assistance.

To our knowledge, this study is the first to use robust methods for analyzing communication in triadic video consultations (ie, among clinician, patient, and informal carer) across multiple clinical settings. Doing so has allowed us to show in detail not only that carers play a vital role in making video consulting work but also how they go about doing this. Our work adds to the existing literature by highlighting the interactional complexity of these consultations, demonstrating the sociotechnical nature of the work undertaken by informal carers, and underscoring the importance of focusing on the microlevel organization of consultations where carers are (and are not) involved [46,50]. Our work was exploratory in nature, relying on secondary analysis; future studies could investigate how the presence of carers affects the overall experience of patients and clinical staff with health care services, the patient-carer relationship, and the health outcomes for patients.

### Conclusions

Video consulting remains a viable service option but depends on patient access to technology and their ability to use it. While many patients can manage a video consultation on their own, some (continue to) require assistance. In these circumstances, informal carers can play a unique, and often invisible, role in making video consultations work. To date, research and guidelines have not adequately considered the work of informal carers. This urgently needs addressing, not only to support the policy vision of the spread of video-consulting services but also to make visible and enable the informal carers (and the patients and clinicians they support) in this often vital role.

## Abbreviations

OTQS: Oxford Telehealth Qualitative Study
SCiP: Supporting Consultations in Remote Physiotherapy
VOCAL: Virtual Online Consultations: Advantages and Limitations

## Data Availability

The data sets generated and analyzed during this study are not publicly available due to confidentiality and sensitivity of the material but are available from the corresponding author on reasonable request.
